# Lack of B and T cell reactivity towards IDH1^R132H^ in blood and tumor tissue from LGG patients

**DOI:** 10.1007/s11060-019-03228-6

**Published:** 2019-06-25

**Authors:** Bas Weenink, Mandy van Brakel, Rebecca Wijers, Peter A. E. Sillevis Smitt, Pim J. French, Reno Debets

**Affiliations:** 1 Department of Neurology, Erasmus MC Cancer Institute, Be 430A, PO Box 2040, 3000 CA Rotterdam, the Netherlands; 2 Laboratory of Tumor Immunology, Department of Medical Oncology, Erasmus MC Cancer Institute, Rotterdam, the Netherlands

**Keywords:** Glioma, Low-grade, IDH1, Neoantigen

## Abstract

**Purpose:**

Mutations in the isocitrate dehydrogenase-1 gene (*IDH1*) occur at high frequency in grade II–III gliomas (LGGs). *IDH1* mutations are somatic, missense and heterozygous affecting codon 132 in the catalytic pocket of the enzyme. In LGG, most mutations (90%) result in an arginine to histidine substitution (IDH1^R132H^) providing a neo-epitope that is expressed in all tumor cells. To assess the immunogenic nature of this epitope, and its potential use to develop T cell treatments, we measured IDH1^R132H^-specific B and T cell reactivity in blood and tumor tissue of LGG patients.

**Methods:**

Sera from IDH1^R132H^-mutated LGG patients (n = 27) were assayed for the presence of a neo-specific antibody response using ELISA. In addition, PBMCs (n = 36) and tumor-infiltrating lymphocytes (TILs, n = 10) were measured for T cell activation markers and IFN-γ production by flow cytometry and ELISA. In some assays, frequencies of CD4 T cells specific for mutated peptide presented by HLA-DR were enriched prior to T cell monitoring assays.

**Results:**

Despite high sensitivity of our assay, we failed to detect IDH1^R132H^-specific IgG in sera of LGG patients. Similarly, we did not observe CD4 T cell reactivity towards IDH1^R132H^ in blood, neither did we observe such reactivity following pre-enrichment of frequencies of IDH1^R132H^-specific CD4 T cells. Finally, we did not detect IDH1^R132H^-specific CD4 T cells among TILs.

**Conclusions:**

The absence of both humoral and cellular responses in blood and tumors of LGG patients indicates that IDH1^R132H^ is not sufficiently immunogenic and devaluates its further therapeutic exploitation, at least in the majority of LGG patients.

**Electronic supplementary material:**

The online version of this article (10.1007/s11060-019-03228-6) contains supplementary material, which is available to authorized users.

## Introduction

Gliomas are the most common primary brain cancers in adults. The WHO currently classifies these tumors based on histological and genetic features into oligodendroglioma, astrocytoma and glioblastoma [1]. Approximately 80% of grade II and III tumors harbor driver mutations in isocitrate dehydrogenase (*IDH*) *1* or *2* genes and are classified as diffuse low-grade gliomas (LGG). Grade IV glioma are classified as high-grade glioma (HGG) and can be distinguished in either primary (IDH wildtype) or secondary (IDH mutant) gliomas [2, 3]. A subset of LGG will progress to HGG within months, while others remain stable for years [4]. Despite advances in neurosurgery, radiotherapy and chemotherapy, almost all glioma patients ultimately die of the disease and thus novel treatment modalities need to be urgently developed.

Recent clinical studies have indicated vaccine- and T cell-based immune therapies as potentially effective novel treatment options for different cancer types [5–8]. For instance, adoptive T cell therapies (ACTs) targeting CD19 have shown durable remissions in patients with refractory B cell ALL and large B cell lymphoma respectively, which has led to FDA approvals of these T cell products to treat B cell malignancies [9, 10]. However, reactivity of therapeutic T cells against healthy tissues has resulted in severe toxicities in recent trials for cancer patients [11–13]. This stressed the importance to select tumor antigens as well as their corresponding chimeric antigen receptors (CARs) or T cell receptors (TCRs) to minimize chances of on- or off-target toxicities [6, 14, 15].

Neoantigens constitute a class of tumor antigens that appear to represent ideal targets for adoptive T cell therapy. These antigens arise from tumor-specific mutations that alter amino acid coding sequences, and hence are not present in any healthy tissues. Different studies have already focused on the therapeutic targeting of neoantigens derived from hallmark glioma mutations, for instance the epidermal growth factor receptor (EGFRvIII), histone H3 (H3.3^K27M^) and isocitrate dehydrogenase 1 (IDH1^R132H^) [16–20]. The IDH1^R132H^ mutation accounts for the vast majority (~ 90%) of all mutations in *IDH1* and results in an arginine to histidine amino acid substitution at codon 132 of this gene [21]. Besides a clear role of this mutant in gliomagenesis through the production of the oncometabolite d-2-hydroxyglutarate [22], the IDH1^R132H^ mutation may provide a unique target for immune therapies as its expression is very frequent, stable and present in all tumor cells [23, 24]. In fact, it has previously been established that IDH1^R132H^ can be presented by HLA-DR, and a spontaneous humoral as well as CD4 T cell response may occur in a subset of glioma patients [20, 25]. In order to develop effective immune therapies targeting IDH1^R132H^, it is critical to assess the frequency and extent of IDH1^R132H^-specific immune reactivity in a cohort of LGG patients. In the current study, we therefore set out to determine the presence of humoral and cellular immune responses directed against IDH1^R132H^, both in peripheral blood and tumor tissue of LGG patients.

## Materials and methods

### Patients and patient samples

Patients with IDH1^R132H^-mutated grade II and III glioma were diagnosed at Erasmus University Medical Center (Rotterdam, The Netherlands). PBMCs and sera were prospectively collected from glioma patients (prior to surgery) and from healthy donors. Formalin-fixed, paraffin-embedded (FFPE) tumor tissue samples were routinely collected for diagnostic purposes. For experiments using TILs, we obtained fresh tissue directly from the operating theatre from suspected LGG patients. The IDH1 mutation status of these tissues was determined following resection by next generation targeted resequencing or immunohistochemistry [23, 26]. In case of unknown IDH1 mutation status, we performed sanger sequencing on DNA isolated from FFPE tumor tissue samples as described previously [27]. All patients provided written informed consent according to national and local regulations for correlative tissue studies. The study was approved by the institutional ethics committee. Patient characteristics are listed in Supplementary table 1.

### HLA-DRB1 typing

HLA-DRB1 typing (low resolution, 2 digit) was performed by Sanquin Diagnostic Services (Amsterdam, the Netherlands) using PCR-SSP on DNA isolated from patient PBMC [28].

### Peptides

Supplementary table 2 shows an overview of used human IDH1^WT^ and IDH1^R132H^ peptides, which were reported to trigger IgG and CD4 T cell reactivity [20]. Negative control peptide used for T cell stimulation assays was human myelin oligodendrocyte glycoprotein. Staphylococcus-derived enterotoxin B (SEB) was used as positive control (Sigma-Aldrich, Zwijndrecht, the Netherlands). Peptides were synthesized by Pepscan (Lelystad, the Netherlands).

### *Anti-IDH1*^*R132H*^* IgG ELISA*

Pierce Streptavidin Coated High Sensitivity Plates (ThermoFisher, Landsmeer, the Netherlands) were washed in a HydroFlex microplate washer (Tecan, Giessen, the Netherlands) using PBS/0.05% Tween20 (Sigma-Aldrich). Plates were coated with 3 µM peptide in PBS/0.05% Tween20 for 1 h. Plates were washed and PBS/0.05% Tween20 + 10% FBS was added to each well (1 h) in order to reduce non-specific antibody binding. Sera from IDH1^R132H^-mutant glioma patients (1:100) were added for 1 h. Plates were washed again and secondary goat anti-human-HRP antibody (1:1000, PI-3000, Vector, Brunschwig chemie, Amsterdam, the Netherlands) was added for 1 h. Primary mouse anti-human IDH1^R132H^ antibody [Clone: H09, 1:5000, Dianova (Bio-Connect, Huissen, the Netherlands)] was used as positive control, to which end, rabbit anti-mouse-HRP antibody (1:1000, P0260, DAKO (Agilent Technologies, Amstelveen, the Netherlands)) was added as second step. After a final wash step, 3,3′,5,5′-tetramethylbenzidine (Sigma-Aldrich) was added and incubated until color developed. Reaction was stopped by addition of 1 M hydrochloric acid, and optical densities (OD) at 450 nm were measured using a Multiskan Ascent Plate Reader (ThermoFisher). All experiments were performed at room temperature.

### PBMCs: isolation, enrichment for peptide-specificity and peptide stimulation

PBMC were isolated from heparin blood by density-gradient centrifugation using Ficoll-Paque PLUS (Sigma-Aldrich). Viable cells and leukocytes were stained using Tuerk solution (Sigma-Aldrich) and Tryptan Blue (ThermoFisher), and microscopically counted. Patient PBMC (2 × 10^6^ cells) were stimulated with 17.5 µM IDH1^R132H^, IDH1^wt^, MOG, or 1 µg/mL staphylococcus-derived enterotoxin B (SEB, Sigma-Aldrich) in 400 µL Iscove’s Modified Dulbecco’s Medium (IMDM) containing 2% l-glutamine, 1% penicillin and streptomycin, supplemented with 6% allogeneic human serum at 37 °C for 16 h.

In some experiments, effector T cells were co-cultured together with peptide-loaded antigen presenting cells prior to peptide stimulation. HeLa cells stably expressing DRA1*01:01 and DRB1*01:01 (DR1 + HeLa) or stably expressing DRA1*01:01 and DRB1*04:01 (DR4 + HeLa) (kind gift from prof. Fred Falkenburg, LUMC, The Netherlands) [29] were irradiated and added to 96 well tissue culture treated plates (Sigma-Aldrich, 0.05 × 10^5^/well) together with autologous patient PBMC (0.4 × 10^5^/well) and 17.5 µM or no peptide in IMDM supplemented with 10 ng/mL IL-7 (200 µL/well). Cells were co-cultured for 3–4 weeks and additional cytokines were added. Day 0: 10 ng/mL IL-7, day 3: 22 ng/mL IL-15, day 6: 100 IU/mL IL-2, day 13: 10 ng/mL IL-7, 1 ng/mL IL-15 and 100 IU/mL IL-2. Cells were split using IMDM containing 360 IU/mL IL-2. Following co-culture, T cells were harvested, washed and assayed (0.2 × 10^6^) for their reactivity upon stimulation with peptide-loaded DR1+, DR4+ or non-transduced HeLa cells (0.2 × 10^6^) at 37 °C for 16 h.

### TILs: isolation and peptide stimulation

TIL microcultures were initiated and expanded from tumor fragments as described previously [30]. In short, suspected LGG tissue was freshly obtained, washed with PBS and cut in small pieces. Single tumor fragments were placed in each well of a 24-well tissue culture plate with 2 mL of Roswell Park Memorial Institute (RPMI) 1640 medium containing 2% l-glutamine, 25 mM HEPES, 1% penicillin and streptomycin, supplemented with 6% allogeneic human serum and 1000 IU/mL IL-2 (TIL medium) at 37 °C. After 2 weeks of culture, contents of wells with clearly visible lymphocyte growth were pooled for each LGG. Viable cells and leukocytes were stained using Tuerk solution (Sigma-Aldrich) and Tryptan Blue (ThermoFisher), and subsequently microscopically counted. Successful cultures of TILs were verified with CD3 flow cytometry (see “Methods” below). To assay reactivity, TILs (2 × 10^6^) were plated with 0.5 × 10^6^ target cells (irradiated autologous PBMC pulsed with 17.5 µM IDH1^R132H^, IDH1^wt^, no peptide) or 1 µg/mL SEB in 200 µL IMDM containing 2% l-glutamine, 1% penicillin and streptomycin, supplemented with 6% allogeneic human serum at 37 °C for 16 h.

### T cell CD137 expression and IFN-γ production

Following T cell stimulations, 2.5 µL PerCP-conjugated mouse anti-human CD45 (BD Biosciences, Vianen, the Netherlands), 5 µL FITC-conjugated mouse anti-human CD3 (BD Biosciences), 5 µL PE-conjugated mouse anti-human CD4 (Beckman Coulter, Woerden, the Netherlands) and 5 µL APC-conjugated mouse anti-human CD137 (BD Biosciences) were premixed in 50 µL PBS and added to cell pellets. Samples were incubated at 4 °C for 30 min, fixed with paraformaldehyde 1% and measured on a FACS Canto (BD Biosciences). Lymphocyte populations were gated using forward scatter (FSC) and side scatter (SSC) plots, and within these lymphocytes CD45+ cells and CD4+ T cells were sequentially gated, after which the latter cells were assessed for CD137 surface expression (Supplementary Fig. 1). Flow cytometric analysis was performed using FCS Express 4 Flow software. Supernatants of T cells following stimulation with peptides or controls were analyzed for the presence of IFN-γ using Human IFN-γ ELISA Ready-SET-Go! kit (ThermoFisher) according to the manufacturer’s instructions.

### Statistics

Statistical analysis was performed using RStudio software and statistical methods used are specified in the figure legends.

## Results

### **No detection of IDH1**^**R132H**^**-specific antibodies in patient serum**

**Fig. 1:**
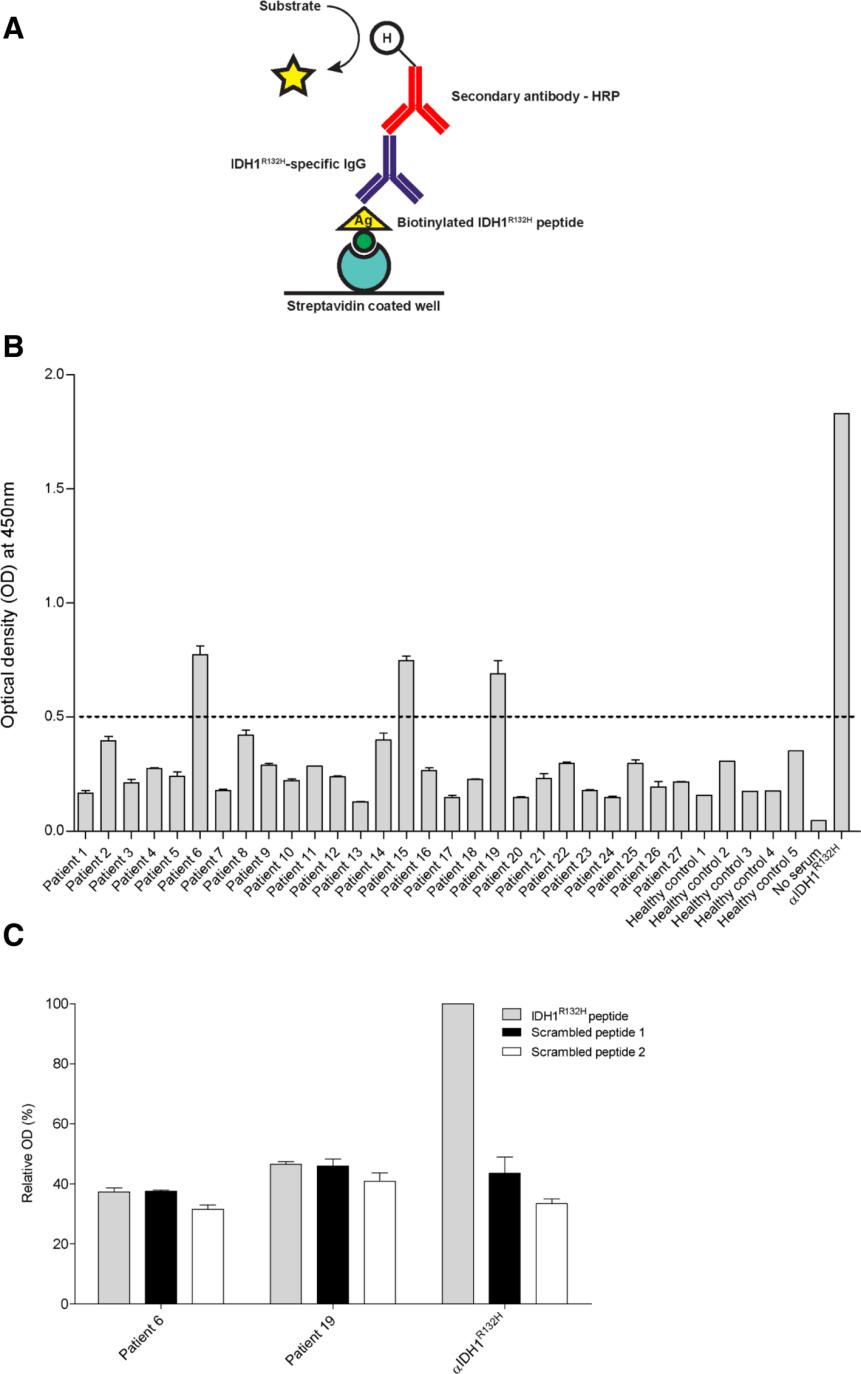
Sera of LGG patients do not contain IDH1^R132H^-specific IgG antibodies. **a** ELISA-based detection scheme of IDH1^R132H^-specific IgG antibodies. **b** Serum samples from IDH1^R132H^-positive LGG patients were screened for mutation-specific IgG antibodies. In 24 out of 27 sera, IDH1^R132H^ peptide-coated wells showed (background) signals comparable to those of healthy controls. Dashed line indicates cut-off for positivity for IDH1^R132H^-specific IgG defined as the mean of healthy control values + 3 × SD. **c** IDH1^R132H^ peptide and scrambled peptide-coated wells were incubated with sera from patients that showed increased optical densities (patients 6 and 19; only samples were re-tested when sufficient sera were available). Same experimental setup was used as in (**a**). Mutant peptide wells did not show increased absorbance when compared to scrambled peptide wells. Assay was performed in triplicate for all 27 patient sera. Data are represented as mean ± SEM. αIDH1^R132H^: primary anti-IDH1^R132H^ antibody, positive control

We first aimed to identify humoral responses targeting the R132H neo-epitope. To this end, we have performed ELISA measurements to detect IDH1^R132H^-specific IgG using sera from LGG patients who harbored the IDH1 mutation (n = 27). Healthy donor sera were included as negative controls (n = 5). Using the experimental setup as depicted in Fig. 1a, we observed that sera of most patients provided ELISA signals comparable to those of negative controls. However, in 3 out of 27 samples (patients 6, 15 and 19), we observed an increased antibody signal towards IDH1^R132H^ peptide (Fig. 1b). Given the fact that both IDH1^R132H^ and scrambled peptides revealed equal signals for these patients (Fig. 1c), these signals were interpreted as non-specific. These findings argue that no IDH1^R132H^-specific antibodies are present in serum of 27 IDH1^R132H^-mutant LGG patients.

### No detection of IDH1^R132H^-specific T cells in patient blood

**Fig. 2:**
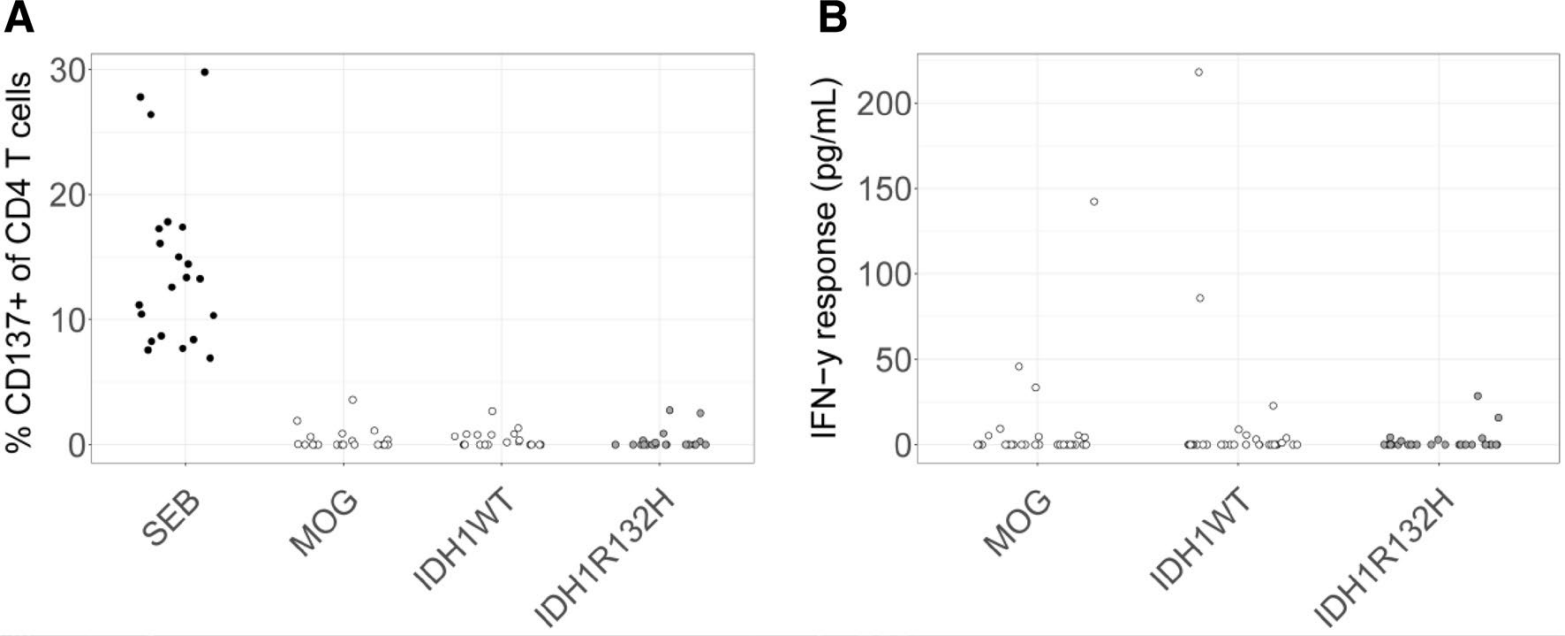
Blood of LGG patients do not harbor CD4 T cell reactivity against IDH1^R132H^. PBMCs from patients with IDH1^R132H^-positive gliomas were directly stimulated with IDH1^R132H^ or IDH1^WT^ peptide for 16 h (n = 30 patients). **a** Percentage of CD137+ cells within CD4+ T cells. **b** IFN-γ response of the same T cell populations as in (**a**). Each dot represents an individual patient sample. MOG, IDH1^WT^, and IDH1^R132H^ were corrected for the medium values. Medium, no peptide, negative control. SEB, staphylococcus-derived enterotoxin B, positive control. MOG, myelin oligodendrocyte glycoprotein peptide, negative control

Next, we screened IDH1^R132H^-mutant glioma patients’ blood for T cell reactivity against this mutant peptide. For this, PBMCs, that contain a mixture of antigen presenting cells and effector T cells, were derived from 30 LGG patients and loaded with IDH1^R132H^ peptide or control peptides, and after a short-term stimulation, T cells were assessed for up-regulated surface expression of CD137 and production of IFN-γ, both measures of TCR-mediated activation. As the used IDH1^R132H^ peptide has been reported to be promiscuous with respect to MHC class II, particularly HLA-DR alleles, no pre-selection of LGG patients was performed based on HLA alleles [20]. None of 30 patient PBMC samples stimulated with IDH1^R132H^ peptide showed an increased frequency of CD4 T cells expressing CD137 compared to non-mutated peptide (Fig. 2a). Supplementary Fig. 1 shows the gating strategy used in this experiment. No increased frequency of CD137-positive CD8 T cells was observed in mutant peptide stimulation conditions either (data not shown). With respect to IFN-γ, measured in supernatants from the same samples, again no enhanced responses were observed in IDH1 mutant versus wildtype peptide stimulations (Fig. 2b).

**Fig. 3:**
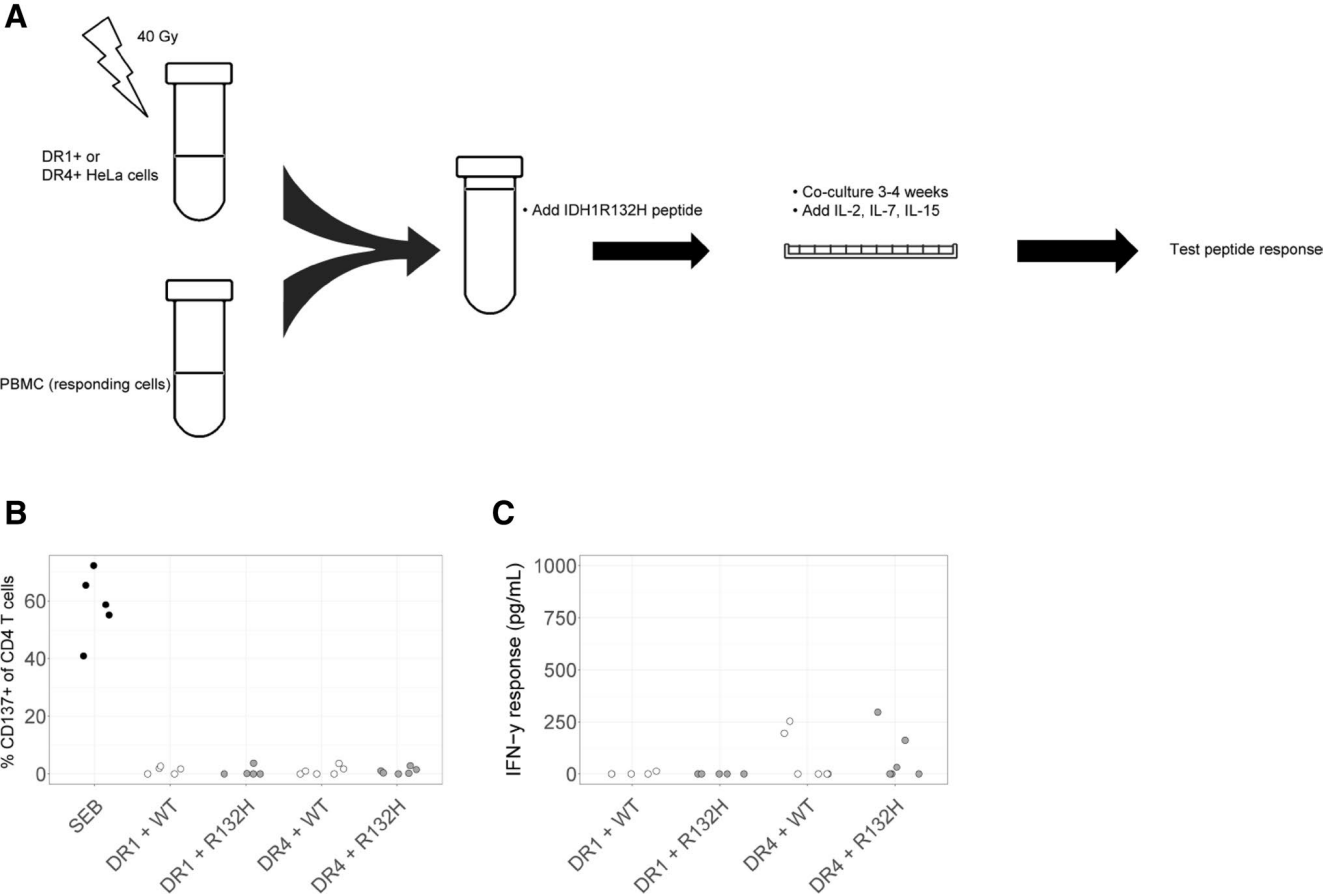
No CD4 T cell reactivity against IDH1^R132H^ despite long-term pre-culture of CD4 T cells in presence of antigen. **a** Scheme of co-culture system used to enrich frequencies of mutant-specific T cells. In short, HLA-DR1 or DR4-positive patient PBMC were co-cultured with HLA-DR1 or -DR4 transduced HeLa cells loaded with IDH1^R132H^ peptide or no peptide for 4 weeks. Prior to measurements of T cell activation, T cell populations (n = 6 patients) were stimulated with DR1 or DR4-positive HeLa cells loaded with IDH1^R132H^ or IDH1^WT^ peptide for 16 h. **b** Percentage of CD137+ cells of CD4+ T cells. **c** IFN-γ response of the same T cell populations used in (**b**). Each dot represents an individual patient sample. DR1 + R132H, DR1 + WT, DR4 + R132H, DR4 + WT were corrected for the medium values. DR1, HeLa cells transduced with DRB1*01:01. DR4, HeLa cells transduced with DRB1*04:01

Frequencies of peripheral T cells directed against tumor antigens are generally very low in patients [31, 32]. For instance, frequencies of neoantigen-specific T cells in melanoma have been reported to range from 0.002% to at most 0.4% of PBMCs [33]. To address this challenge, we set up a co-culture system to enrich the frequency of IDH1^R132H^ peptide-specific T cells prior to analyzing peptide reactivity. We have previously shown the feasibility of pre-enrichment of antigen-reactive T cells without loss of T cell specificity [29, 34, 35]. In the current study, we have co-cultured patient PBMC with HeLa cells stably expressing HLA-DR1 or DR4, two alleles that were described to facilitate IDH1^R132H^ peptide-specific T cell responses [20], and loaded with IDH1^R132H^ peptide (Fig. 3a). Following 3–4 co-cultivation cycles, T cells were re-stimulated with peptide-loaded HeLa cells, yet again no consistent effect of mutated peptide versus wild-type peptide towards T cell activation was observed in 6 out of 6 patient samples (Fig. 3b, c). Collectively, when using PBMC from LGG patients who harbor the IDH1 mutant, whether or not pre-enriched for T cell reactivity against mutant IDH1 peptide, we did not observe IDH1^R132H^-specific CD4 T cell responses.

### No detection of IDH1^R132H^-specific T cells in tumor tissue

**Fig. 4:**
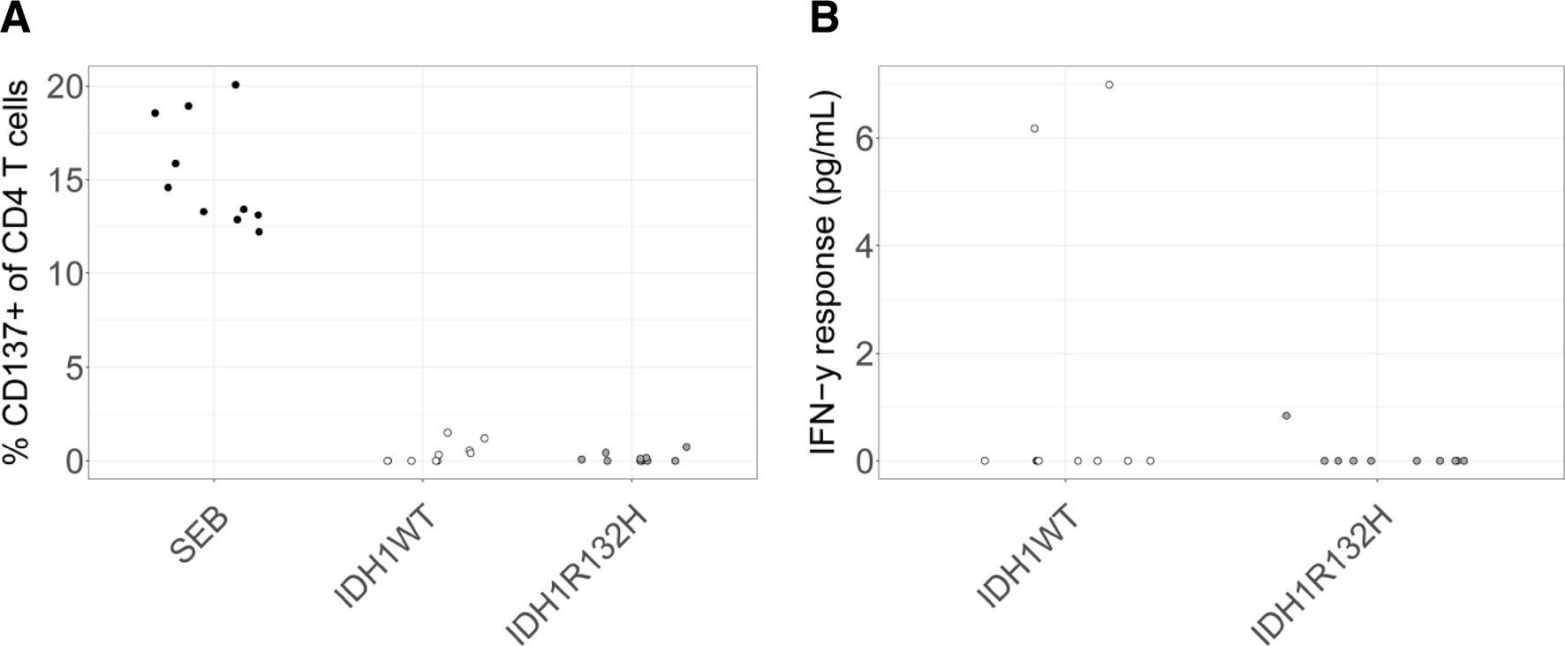
Tumors of LGG patients do not harbor CD4 T cell reactivity against IDH1^R132H^. TILs were obtained as described and expanded from fresh LGG tumor fragments in IL-2 supplemented medium during 2 weeks. TILs were then stimulated with IDH1^R132H^ or IDH1^WT^ peptide-loaded, irradiated autologous PBMC for 16 h (n = 10 patients). **a** Percentage of CD137+ cells of CD4+ T cells. **b** IFN-γ response of the same TIL populations as in (**a**). Each dot represents an individual patient sample. IDH1^R132H^ and IDH1^WT^ were corrected for the medium values

When compared to blood, the number of mutant-specific T cells may be enriched in IDH1^R132H^ expressing tumor tissue in case an effective T cell response had occurred [36–39], providing a rationale to screen for the presence of IDH1^R132H^-specific CD4 T cells among TILs. TILs were polyclonally expanded from fresh resection material derived from 10 LGGs (with IDH1 mutation) and were subsequently stimulated with IDH1^R132H^ peptide loaded, irradiated autologous PBMCs. Also using TILs, we were not able to observe changes with respect to CD137 expression and IFN-γ production of CD4 T cells for 10 out of 10 patients (Fig. 4).

## Discussion

In this study, we focused on detecting the presence of IDH1^R132H^-specific T and B cell reactivity in IDH1^R132H^-mutant LGG patients. Detection of immune reactivity against this mutated peptide would warrant and at the same time facilitate the development of immunotherapy towards IDH1^R132H^+ gliomas. Using various readouts for B and T cell responses, and using sera (n = 27 patients), PBMC (n = 30) as well as TILs (n = 10), we showed that immune cell populations with reactivity towards mutant IDH1 are not present in the LGG patient samples studied here.

These findings are in contrast to previous observations where Schumacher and colleagues demonstrated humoral and CD4 T cell responses in patients with glioma [20]. In our study we cannot exclude that we were not able to detect mutant-specific immune reactivity despite their presence in (a subset of) LGG patients and this could be due to a number of reasons.

First, levels of IDH1^R132H^-specific antibodies or frequencies of IDH1^R132H^-specific CD4 T cells may be below the detection thresholds of assays. For instance, among TILs from (multiple and pooled) IDH1^R132H^-mutant tumors, the frequency of IDH1^R132H^-specific CD4 T cells was less than 2% (of CD4+ T cells) after vaccinating mice with mutant peptides [20]. Indeed, the central nervous system is considered to be immune-privileged, e.g. due to limited MHC expression, and consequently elicitation of robust glioma-specific immune responses may be limited [40]. Along this line, suppression of anti-tumor T cell immunity by the oncometabolite d-2-hydroxyglutarate has also recently been described in LGG [41], which may further limit local activation of IDH1^R132H^-specific CD4 T cells. A tentative low frequency of IDH1^R132H^-specific CD4 T cells was addressed by pre-enrichment of these cells by co-culturing PBMC of IDH1^R132H^-mutant patients with HeLa cells presenting IDH1^R132H^. None of the samples showed a IDH1^R132H^-specific T cell activation response, whether or not CD4 T cells underwent co-culture cycles with IDH1^R132H^-presenting cells [42, 43]. Although the occurrence of very low frequencies of IDH1^R132H^-specific T cells cannot be dismissed, and these could be targeted with enrichment protocols using more professional antigen-presenting cells, optimal pMHC bindings [44] or in vivo vaccinations with IDH1^R132H^ [45], our data do suggest that the immunogenicity of IDH1^R132H^ is low.

Second, the number of patients assayed may have been too small in order to detect a cellular (n = 46; 36 PBMC and 10 TIL samples) or humoral immune response (n = 27). Percentages of patients with antibody or CD4 T cell responses in blood have been reported to be as low as 10% and 16%, respectively [20]. However, even in the case of a population frequency of 10%, there is a 94% chance of detecting at least one positive humoral response, and a 75% chance if the population frequency is 5% (using a cohort of 27 samples). Moreover, when combining the 36 PBMC and 10 TIL samples used to detect T-cell responses against IDH1^R132H^, there is > 99% or 91% chance of detecting such responses in population frequencies of at least 10% or 5% respectively.

Third, we cannot exclude that IDH1^R132H^ may be presented by specific HLA alleles that are underrepresented in our cohort of tested patients and/or that expression levels of *IDH1 *are too low to elicit IDH1^R132H^-specific T-cell responses. Indeed, glioblastomas express *IDH1* at higher levels compared to gliomas of lower grade (log2 expression levels of 10.9 ± 0.48 vs. 11.3 ± 0.49, vs. 11.4 ± 0.48 for grade II (n = 24), grade III (n = 85) and grade IV gliomas (n = 159) respectively [46]). However, the relative increase in IDH1 expression with increasing tumor grade is very modest and expression levels of IDH1 are in general relatively high. Moreover, there is no difference between grade III astrocytomas and grade IV glioblastomas 11.4 ± 0.48 vs. 11.4 ± 0.43, p = 0.89).

Taken together, our data does not provide evidence for detectable presence of immune cell reactivity towards IDH1^R132H^ in blood or tumors of LGG patients. We advocate further preclinical studies prior to development and clinical exploitation of T cell treatments directed against IDH1^R132H^ in LGG patients.

## Electronic supplementary material

Below is the link to the electronic supplementary material.

Supplementary file1 (DOCX 180 kb Supplementary table 1. Patient characteristics and analyses. Histopathological diagnosis and IDH1R132H mutation states are shown for all LGG patient samples used in this study, as well as HLA-DRB1 allele usage. IDH1R132H glioma patient serum was used for IgG ELISA, patient PBMCs and TILs were used for direct T cell stimulation assays. In some cases patient PBMCs were used to pre-enrich frequencies of IDH1R132H-specific T cells (bold); n.d., not determined. Supplementary table 2. Peptides used in functional assays. Supplementary fig. 1. Gating strategy to assess frequency of IDH1R132H-specific CD4+ T cells. Using flow cytometry, viable lymphocyte populations were first gated on FSC/SSC, after which CD45+ cells were gated and CD137 surface expression was assessed within CD4+ T cells.
