# Supplementary material for: Lack of B and T cell reactivity towards IDH1R132H in blood and tumor tissue from LGG patients
Source: J Neurooncol. 2019 Jun 25;144(1):79–87. doi: 10.1007/s11060-019-03228-6 (PMC6660510; doi:10.1007/s11060-019-03228-6)
Supplement: Supplementary file 1 — Supplementary file1 (DOCX 180 kb Supplementary table 1. Patient characteristics and analyses. Histopathological diagnosis and IDH1R132H mutation states are shown for all LGG patient samples used in this study, as well as HLA-DRB1 allele usage. IDH1R132H glioma patient serum was used for IgG ELISA, patient PBMCs and TILs were used for direct T cell stimulation assays. In some cases patient PBMCs were used to pre-enrich frequencies of IDH1R132H-specific T cells (bold); n.d., not determined. Supplementary table 2. Peptides used in functional assays. Supplementary fig. 1. Gating strategy to assess frequency of IDH1R132H-specific CD4+ T cells. Using flow cytometry, viable lymphocyte populations were first gated on FSC/SSC, after which CD45+ cells were gated and CD137 surface expression was assessed within CD4+ T cells. [file 11060_2019_3228_MOESM1_ESM.docx]

**Supplementary table 1**

| **Gender** | **Diagnosis** | **Previous therapies** | **Resection status** | **IDH1^R132H^** | **HLA-DRB1** | **Serum patient #** | **PBMC patient #** | **TIL patient #** |
| --- | --- | --- | --- | --- | --- | --- | --- | --- |
| F | Oligodendroglioma (grade II) | STX, PVC | b | Yes | n.d. | 1 |  |  |
| F | Astrocytoma (grade III) | S, RCHT (TMZ), S | c, c | Yes | n.d. | 2 |  |  |
| F | Astrocytoma (grade II) | S, RT | c | Yes | n.d. | 3 |  |  |
| F | Astrocytoma (grade II) | S, S, RT, TMZ, LOM | c, c | Yes | n.d. | 4 |  |  |
| M | Oligodendroglioma (grade II) | S | c | Yes | n.d. | 5 |  |  |
| M | Astrocytoma (grade II) | STX, S, RCHT (TMZ) | b, p | Yes | n.d. | 6 |  |  |
| M | Astrocytoma (grade II) | S, RCHT (TMZ), S, TMZ, LOM | c, p | Yes | n.d. | 7 |  |  |
| M | Oligodendroglioma (grade III) | S, RT, PCV | p | Yes | n.d. | 8 |  |  |
| M | Astrocytoma (grade III) | S, TMZ, RT, S, RT | c, p | Yes | n.d. | 9 |  |  |
| F | Oligoastrocytoma (grade III) | S, RCHT (TMZ), RCHT (TMZ), S, LOM | p, p | Yes | n.d. | 10 |  |  |
| M | Astrocytoma (grade II) | S, RT, TMZ | p | Yes | n.d. | 11 |  |  |
| F | Astrocytoma (grade III) | S, S, RT, PCV | c, c | Yes | n.d. | 12 |  |  |
| M | Oligoastrocytoma (grade III) | S, RT | p | Yes | n.d. | 13 |  |  |
| M | Oligodendroglioma (grade III) | S, PCV | p | Yes | n.d. | 14 |  |  |
| M | Astrocytoma (grade III) | S, S, RCHT (TMZ), S, TMZ, LOM | c, p, p | Yes | n.d. | 15 |  |  |
| M | Astrocytoma (grade II) | STX, RCHT (TMZ), S, S, TMZ | b, p, p | Yes | n.d. | 16 |  |  |
| F | Astrocytoma (grade III) | S, RCHT (TMZ) | p | Yes | n.d. | 17 |  |  |
| F | Astrocytoma (grade II) | S | p | Yes | n.d. | 18 |  |  |
| M | Oligodendroglioma (grade III) | S, RCHT (TMZ) | p | Yes | n.d. | 19 |  |  |
| M | Astrocytoma (grade III) | S, RCHT (TMZ), LOM, RT | c | Yes | n.d. | 20 |  |  |
| M | Oligodendroglioma (grade II) | S, RT, PCV | b | Yes | n.d. | 21 |  |  |
| F | Astrocytoma (grade III) | S, RT | p | Yes | n.d. | 22 |  |  |
| M | Astrocytoma (grade II) | STX, RT, S, TMZ, LOM | b, p | Yes | DRB1*03 | 23 | 1 |  |
| M | Oligoastrocytoma (grade III) | S, RCHT (TMZ), LOM | c | Yes | n.d. |  | 2 |  |
| M | Oligodendroglioma (grade III) | S, RCHT (PCV) | c | Yes | DRB1*03 DRB1*04 |  | 3, ***1*** |  |
| V | Astrocytoma (grade III) | STX, S, RCHT (TMZ) | b, p | Yes | DRB1*14 DRB1*15 | 24 | 4 |  |
| M | Oligodendroglioma (grade II) | S, PCV | c | Yes | DRB1*03 DRB1*15 |  | 5 |  |
| M | Oligodendroglioma (grade II) | S, PCV, RT | p | Yes | DRB1*11 DRB1*15 |  | 6 |  |
| M | Astrocytoma (grade II) | S, RT, PCV, TMZ | p | Yes | DRB1*07 DRB1*13 |  | 7 |  |
| M | Oligodendroglioma (grade III) | S, RT, PCV, TMZ | p | Yes | DRB1*04 DRB1*11 |  | 8 |  |
| V | Oligodendroglioma (grade III) | S, S, RCHT (TMZ) | c, c | Yes | DRB1*03 DRB1*09 |  | 9 |  |
| M | Oligodendroglioma (grade II) | STX, RT, PCV | b | Yes | n.d. |  | 10 |  |
| V | Oligodendroglioma (grade II) | S, RT | c | Yes | DRB1*01 DRB1*04 |  | 11 |  |
| M | Oligodendroglioma (grade II) | S | c | Yes | DRB1*14 DRB1*16 |  | 12 |  |
| M | Oligodendroglioma (grade III) | S, RT, PCV | p | Yes | DRB1*13 DRB1*15 | 25 | 13 |  |
| M | Astrocytoma (grade II) | STX, TMZ | b | Yes | DRB1*04 DRB1*15 | 26 | 14 |  |
| M | Oligodendroglioma (grade III) | S, PCV, TMZ, RT, LOM, S, S | c, p, p | Yes | DRB1*07 DRB1*10 |  | 15 |  |
| M | Oligodendroglioma (grade III) | S, RT, S | c, c | Yes | DRB1*13 |  | 16 |  |
| M | Astrocytoma (grade III) | S | p | Yes | DRB1*01 DRB1*04 |  | 17 |  |
| M | Oligodendroglioma (grade II) | S, PCV | c | Yes | DRB1*01 DRB1*03 | 27 | 18 |  |
| M | Astrocytoma (grade III) | S, RT | c | Yes | DRB1*01 DRB1*15 |  | 19 |  |
| V | Oligodendroglioma (grade II) | S | p | Yes | DRB1*04 |  | 20 |  |
| M | Astrocytoma (grade III) | S, RCHT (TMZ) | c | Yes | DRB1*07 DRB1*10 |  | 21 |  |
| M | Astrocytoma (grade II) | S, RT, TMZ, RT | p | Yes | n.d. |  | 22 |  |
| V | Astrocytoma (grade II) | S | p | Yes | DRB1*07 DRB1*11 |  | 23 |  |
| M | Astrocytoma (grade II) | S, S, RT | p, p | Yes | DRB1*01 DRB1*03 |  | 24 |  |
| M | Astrocytoma (grade II) | S | p | Yes | DRB1*04 DRB1*11 |  | 25 |  |
| V | Oligoastrocytoma (grade II) | S, S | c, p | Yes | DRB1*04 DRB1*12 |  | 26 |  |
| M | Oligodendroglioma (grade II) | S | c | Yes | DRB1*01 DRB1*08 |  | 27 |  |
| V | Astrocytoma (grade III) | S | p | Yes | DRB1*04 DRB1*15 |  | 28 |  |
| M | Oligodendroglioma (grade II) | S, PCV | p | Yes | DRB1*03 DRB1*16 |  | 29 |  |
| V | Astrocytoma (grade II) | S | p | Yes | n.d. |  | 30 |  |
| M | Astrocytoma (grade III) | S, RT | p | Yes | DRB1*01 DRB1*14 |  | ***2*** |  |
| F | Oligodendroglioma (grade II) | STX, PCV | b | Yes | DRB1*04 DRB1*12 |  | ***3*** |  |
| M | Oligoastrocytoma (grade III) | STX | b | Yes | DRB1*07 DRB1*04 |  | ***4*** |  |
| V | Oligodendroglioma (grade III) | S, RT, TMZ, S, TMZ, S | p, p, p | Yes | DRB1*03 DRB1*01 |  | ***5*** |  |
| M | Oligoastrocytoma (grade II) | S, RT, TMZ, S, S, RT | c, c, p | Yes | DRB1*03 DRB1*01 |  | ***6*** |  |
| M | Oligoastrocytoma (grade III) | S, RT, S | p, c | Yes | n.d. |  |  | 1 |
| F | Astrocytoma (grade II) | S | p | Yes | n.d. |  |  | 2 |
| F | Oligoastrocytoma (grade II) | S | p | Yes | n.d. |  |  | 3 |
| M | Astrocytoma (grade II) | S | c | Yes | n.d. |  |  | 4 |
| M | Oligodendroglioma (grade II) | S | c | Yes | n.d. |  |  | 5 |
| F | Oligodendroglioma (grade II) | S | c | Yes | n.d. |  |  | 6 |
| F | Oligodendroglioma (grade II) | S | p | Yes | n.d. |  |  | 7 |
| M | Astrocytoma (grade II) | S | p | Yes | n.d. |  |  | 8 |
| M | Astrocytoma (grade III) | S | p | Yes | n.d. |  |  | 9 |
| M | Oligodendroglioma (grade II) | S | c | Yes | n.d. |  |  | 10 |

| **Gender** |  | **Diagnosis** | **IDH1^R132H^+** | **HLA-DRB1** | **Serum patient #** | **PBMC patient #** | **TIL patient #** |
| --- | --- | --- | --- | --- | --- | --- | --- |

S, surgery; RT, radiotherapy; RCHT, radiochemotherapy; TMZ, temozolomide; PCV, procarbazine; LOM, lomustine; BEV, bevacizumab; STX, stereotactic biopsy; n.d., not determined; b, biopsy; p, partial; c, complete.

**Supplementary table 2**

| **Assay** | **Peptide** | **Amino acid sequence** |
| --- | --- | --- |
| IgG ELISA | IDH1^WT^ p122–136 | SGWVKPIIIGRHAYG |
|  | IDH1^R132H^ p122–136 | SGWVKPIIIGHHAYG |
|  | IDH1^WT^ p123–142 | GWVKPIIIGRHAYGDQYRAT |
|  | IDH1^R132H^ p123–142 | GWVKPIIIGHHAYGDQYRAT |
|  | Scrambled peptide 1 | DHPSVPCFLDRWD |
|  | Scrambled peptide 2 | CDNSGEIYGYICRY |
| T cell stimulations | IDH1^WT^ p123–142 | GWVKPIIIGRHAYGDQYRAT |
|  | IDH1^R132H^ p123–142 | GWVKPIIIGHHAYGDQYRAT |
|  | MOG p35–55 | IGPRHPIRALVGDEVELPCR |

**Supplementary fig. 1**

**
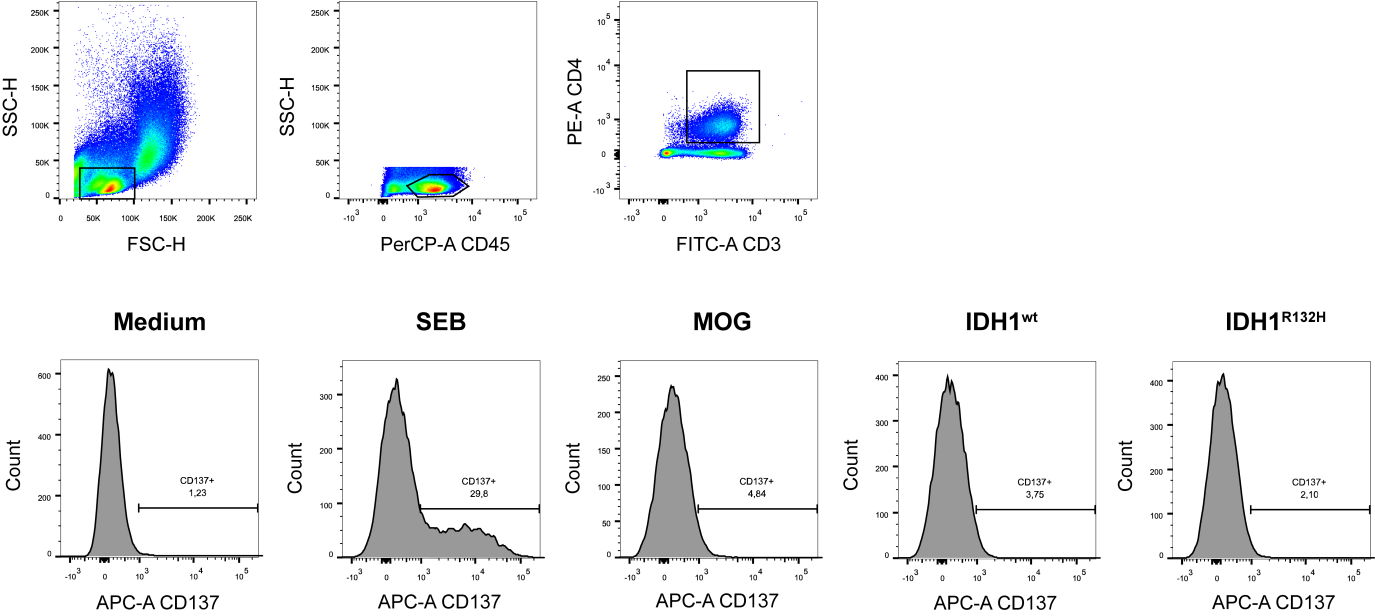
**
